# Food Consumption Frequency Based on the Mini Nutritional Assessment (MNA) and Its Association with Probable Sarcopenia as Measured by Handgrip Strength in a Group of Chilean Older Persons Aged 65 and over

**DOI:** 10.3390/nu17111773

**Published:** 2025-05-23

**Authors:** Camila Henríquez Mella, Mirta Crovetto

**Affiliations:** Departamento de Salud, Comunidad y Gestión, Facultad de Ciencias de la Salud, Universidad de Playa Ancha, Valparaíso 2340000, Chile; camila.henriquez@upla.cl

**Keywords:** aging, sarcopenia, muscle strength, nutrition assessment, feeding behavior

## Abstract

**Background/Objectives:** Sarcopenia, characterized by the loss of muscle mass and strength, is prevalent in older persons and affects their quality of life. Nutritional intervention and physical activity play a key role in its prevention and treatment. This study aims to investigate the relationship between food consumption frequency as assessed through the Mini Nutritional Assessment (MNA) and probable sarcopenia, evaluated by grip strength, in Chilean older persons aged 65 or older. **Methods:** A correlational, cross-sectional study with a non-probabilistic sample of 155 older persons aged 65 or older was undertaken. Food consumption frequency was assessed using the MNA, and muscle strength was measured using a handgrip dynamometer. The authors analyzed the relationship between food consumption frequency, as assessed by the MNA, and the protein intake index with muscle strength. **Results:** Participants who consumed less than two servings of fruits and vegetables per day were 4.28 times more likely to have low muscle strength compared to those who consumed two or more servings per day (OR = 4.28; 95% CI: 1.59–11.45). No significant associations were found with the consumption of dairy products, legumes, meat, fish, poultry, or fluids. The protein intake index did not show a significant relationship with muscle strength. **Conclusions:** The results suggest that a diet rich in fruits and vegetables may have a protective effect on muscle strength in older persons. Promoting adequate intake of these foods could be critical in the prevention of sarcopenia in this population.

## 1. Introduction

The proportion of older persons is increasing globally. In 2019, the number of people aged 60 or older reached 1 billion, a figure that is projected to rise to 1.4 billion by 2030 and 2.1 billion by 2050, with a rapid growth being noted in developing countries [1].

In Chile, this process of population aging is also remarkable. Preliminary results from the 2024 Population and Housing Census in Chile have shown an accelerated demographic aging process. The population aged 65 and older represented 14% of the total, reflecting an increase from the 11.4% recorded in the 2017 Census. Additionally, the Aging Index has reached a value of 79, indicating that for every 100 individuals under 15 years old, there are 79 older persons, compared to 56.9 reported in 2017. In particular, the Valparaíso region has the highest aging index (98.6) [2]. Furthermore, life expectancy has increased significantly, currently standing at 84 years for women and 78 years for men, with projections of 87 and 84 years, respectively, by 2050. These figures reflect a marked demographic aging in the country [3].

Health in older persons is determined by a complex interaction between biological, lifestyle, social, and environmental factors that accumulate over the course of their lives [4]. In Chile, older persons face significant challenges, such as food insecurity, which affects nearly 3 million people in the country, according to data from the Food and Agriculture Organization of the United Nations (FAO) [5]. Additionally, 12% of older persons have reported that their diet is insufficient [6], reflecting that older persons in Chile may be exposed to a higher risk of deterioration in their quality of life.

From a biological perspective, the quality of life of older persons is disrupted by multiple physiological changes associated with aging, such as the reduction in muscle mass, loss of bone tissue, and decreased functional capacity. Additionally, increase in life expectancy is accompanied by a higher prevalence of non-communicable diseases (NCDs), such as diabetes, cardiovascular diseases, obesity, cancer, and neurodegenerative diseases [4]. In this context, optimal dietary patterns play a fundamental role, since a balanced diet, that is adapted to aging-related needs—even when it is incorporated earlier in life—may contribute significantly to the prevention or delay of the onset of NCDs, thus promoting healthy aging [7].

A critical challenge associated with aging is sarcopenia, a progressive and generalized skeletal muscle disorder that increases the risk of falls, fractures, physical disability, and mortality. Sarcopenia can be primary, attributed to aging, or secondary, linked to systemic diseases, physical inactivity, or malnutrition [8].

Nutritional status and sarcopenia are closely related. Sarcopenia can occur as a result of inadequate energy or protein intake, which may be due to anorexia, malabsorption, limited access to healthy foods, or limited ability to eat [8], contributing to a progressive loss of muscle mass and muscle function, which are a feature of sarcopenia. Therefore, maintaining an optimal nutritional status is essential to prevent or delay the onset of sarcopenia.

Recent evidence has shown a direct relationship between nutrient intake and sarcopenia. For instance, a systematic review and meta-analysis has shown that older persons with sarcopenia had a significantly lower protein intake than their peers without this condition [9]. Similarly, another systematic review showed that older persons with sarcopenia have a significantly lower consumption of calories, proteins, carbohydrates, saturated fatty acids, and essential nutrients such as vitamins A, B12, C, and D, as well as minerals like calcium, magnesium, sodium, and selenium, compared to those without this condition [10]. Additionally, a higher consumption of fruits and vegetables has been associated with a significant reduction in the risk of developing sarcopenia [11].

In this context, tools such as the MNA have been validated to assess the nutritional status of older persons in an effective and practical way in various settings. This instrument, designed to detect malnutrition and the risk of malnutrition, facilitates early and timely nutritional interventions [12,13,14,15].

Similarly, the 2019 European Working Group on Sarcopenia in Older People 2 (EWGSOP2) Consensus identified muscle strength as a key parameter in the diagnosis of sarcopenia, with grip strength being a simple and reliable method for its assessment. Low grip strength is associated with worse clinical outcomes, including higher hospitalization rates, functional decline, lower quality of life, and increased mortality [8].

In Chile, although malnutrition and its risk factors have been studied in the elderly population, limited research has been conducted on the relationship between diet and muscle strength, particularly regarding sarcopenia. This knowledge gap hinders the development of effective nutritional interventions for this growing and vulnerable population, highlighting the need for research that addresses these challenges and leads to more efficient public policies, reducing the burden of chronic diseases and associated healthcare costs.

In this context, the present research aims to study the relationship between food consumption frequency, assessed through the MNA, and probable sarcopenia, measured through grip strength, in a group of Chilean older persons aged 65 and older.

## 2. Materials and Methods

Design and Participants: This was a correlational, cross-sectional study, corresponding to a subsample of Chilean older persons from the 2018 Multicenter Study on Nutrition in Older Persons (NAM). The study involved Chilean older persons (men and women aged 65 or older), both institutionalized and non-institutionalized. The institutionalized group included residents of geriatric centers in Long-term Care Institutions for Older Persons, which operate under the guidance of social policies in Chile. The non-institutionalized group consisted of individuals engaged in older persons organizations, residents of Assisted Housing Facilities, members of neighborhood councils, or private individuals. The researchers used a non-probabilistic convenience sampling method.

Participants who were able to carry out their usual activities without restrictions due to health problems and who signed an informed consent to participate in the assessments process on the intervention day were included. The initial sample consisted of 171 older persons. Those persons with physical and/or psychological conditions that prevented them from responding to and/or performing the activities included in the study were excluded, based on the information provided by the participants themselves or their caregivers. After applying exclusion criteria, the final sample consisted of 155 older persons. For the purposes of this study, an older person was defined as a person aged 65 or older, according to the criteria of the Ministry of Health (MINSAL) of Chile.

Probable sarcopenia (as measured by grip strength through dynamometry) was defined as the dependent variable, and food consumption frequency based on the MNA was defined as the independent variable.

Data Collection: This was conducted between August and December 2018, led by nutritionists from the Faculty of Health Sciences at the University of Playa Ancha. The evaluation included muscle strength testing through dynamometry, the application of the MNA during an interview, anthropometric measurements (weight, height, arm and calf circumference), and gait speed test. Data were immediately recorded in predefined formats. The specific procedures for data collection, nutritional, and anthropometric evaluation have been previously described in detail [16]. It should be noticed that, although the original study gathered sufficient information to apply the three diagnostic criteria proposed by the EWGSOP2 (muscle strength, muscle mass, and physical performance), our analysis only considered one subset of these data, focused on the relationship between probable sarcopenia as assessed by grip strength and food consumption frequency.

Probable sarcopenia: For the purposes of the baseline study, the diagnosis of sarcopenia was based on the EWGSOP2 algorithm, which includes three criteria: (1) low muscle strength, measured by grip strength; (2) low muscle mass or quantity, assessed by calf circumference (CC); and (3) low physical performance, determined by the gait speed test. In this study, probable sarcopenia was identified when criterion 1 was exclusively met; that is, a significant decrease in grip strength was observed. Diagnostic confirmation of sarcopenia required both criterion 1 and criterion 2, while severe sarcopenia was diagnosed when all three criteria were met [8].

Muscle strength: This was evaluated by measuring grip strength using a JAMAR^®^ hydraulic dynamometer (0–200 pounds or 90 kg, 5 positions). The dominant hand was used, and two attempts were considered, with the best value recorded. Low muscle strength was defined as a cutoff of <27 kg for men and <15 kg for women, according to previously used values which were described in this same sample in a previous research study [16]. Similarly, the procedure for conducting measurements has been previously described in detail [16].

Food consumption frequency according to MNA. The MNA test is structured into four parts: anthropometric assessment (weight loss, body mass index (BMI), and body circumferences); general assessment (lifestyle, medication use, and mobility); dietary assessment (number of meals, food and fluid intake, autonomy to eat); and self-assessment (self-perception of health and nutrition) [17]. To determine the frequency of food consumption, dietary assessment was conducted using the MNA, which includes 5 questions related to food: (i) number of meals; (ii) protein intake (dairy, legumes or eggs, meat, fish, or poultry); (iii) fruits and vegetables; (iv) fluids (water, juice, coffee, tea, or milk); and (v) autonomy to eat. Regarding the questions about the frequency of food consumption and portions by food groups, definitions were previously established following the instructions according to the guidelines for completing the MNA [18].

To determine the number of meals in a day, a meal was defined as the intake of more than two food items or preparations when the subject sits down to eat. Each participant was asked to name all the meal times they had, with the following options: breakfast, lunch, tea time, dinner, or snack. One portion of dairy products was defined as “a glass of milk”, “cheese in a sandwich”, or “a yogurt”. One portion of legumes was equal to one cup of cooked legumes, and one portion of eggs was equivalent to one unit. Portions of meat, fish, or poultry were defined as a steak the size of the palm of the hand. For fruits and vegetables, one portion was equivalent to one medium piece or a medium glass of fruit juice, and one cup of raw or cooked vegetables. Finally, one portion of fluids was defined as a 200–250 mL cup of water or other fluids such as infusions, coffee, milk, juices, fruit juices, or other liquid food products.

Protein intake index: The protein intake index was calculated based on the score obtained in the MNA section corresponding to protein intake. This section included three key questions: (1) Do you consume at least one portion of dairy products per day? (milk, cheese, yogurt); (2) Do you consume two or more portions of legumes or eggs per week? and (3) Do you consume meat, fish, or poultry on a daily basis? Each affirmative answer was given one point, yielding a total score ranging from 0 to 3. Based on these results, three levels were established to describe protein intake index: Good (3 affirmative answers), Regular (2 affirmative answers), and Low (0 to 1 affirmative answer).

Statistical analysis: Data analysis was performed using the Stata 17 statistical software (StataCorp, College Station, TX, USA). Differences in muscle strength according to the frequency of consumption of food groups (dairy products, legumes or eggs, meat, fish, or poultry, fruits and vegetables, and fluids) were analyzed using Student’s *t*-test or ANOVA, as appropriate. To compare muscle strength across protein index levels (good, regular, or low), an ANOVA was used. The association between food consumption frequency and muscle strength (normal vs. low) was evaluated using the Chi-square test. Finally, a logistic regression analysis was performed to examine the association between low muscle strength and various nutritional and sociodemographic variables. The following nutritional variables were considered: daily consumption of dairy products; legumes or eggs; meat, fish, or poultry; fruits and vegetables; and fluid intake. Additionally, the protein index, categorized as good, regular, or low, was included. The following sociodemographic variables were considered: nutritional status according to MNA, gender, and age. Low muscle strength was defined as the dependent variable in the regression. Variables were adjusted for gender and age, with an additional analysis evaluating the association between food group consumption frequency and muscle strength. A *p*-value of <0.05 was considered as the significance threshold, and associations were expressed through odds ratios (OR) and 95% confidence intervals (95% CI).

The study was developed in accordance with the Declaration of Helsinki on research including human subjects. The study was approved on 3 April 2018 by the Research Ethics Committee of the Universidad de Maimónides, Argentina. Older persons voluntarily agreed to participate, and each participant signed the informed consent, which was based on the protocol of the 2018 Multicentric Study on Nutrition in Older Persons (NAM).

## 3. Results

The sample consisted of 155 participants, with a mean age of 77.1 ± 7.1 years. Most participants were women (72.3%), and 61.3% were over 75 years old. Of the total sample, only about 10% were institutionalized. In terms of nutritional status according to BMI, the majority of the sample (52.9%) was classified as malnourished due to obesity and overweight. According to the MNA, 28.4% were at risk of malnutrition, and a small percentage (5.2%) were malnourished. Physical measurements, including mean values for brachial circumference, calf circumference, and muscle strength, are shown in Table 1.

Men had significantly higher weight, height, and muscle strength than women (*p* < 0.01). No differences were observed according to sex in BMI or Nutritional Status according to MNA.

Regarding food intake, 72.9% reported having a normal food intake; that is, there was no decrease in the last three months, while 26% reported eating less or much less. The majority (95.2% of participants) reported eating by themselves and without difficulty. Regarding the number of complete meals per day, 96.8% of participants consume three meals a day. Regarding the frequency of food consumption, 60% reported consuming at least one portion of dairy products per day, while 40% did not reach this frequency. The 85.8% reported consuming at least two portions of legumes or eggs per week, and 58.7% consume meat, fish, or poultry on a daily basis. Most participants (76.8%) consume at least two portions of fruits or vegetables per day, while 23.2% do not reach this frequency. More than half of the participants (52.3%) consume more than five cups of fluids per day, while 38.1% consume between three and five cups, and 9.7% reported insufficient consumption (<3 cups per day). No significant differences were found according to sex for any of these variables (Table 2).

Table 3 shows differences in muscle strength according to the frequency of consumption of the different food groups analyzed. Regarding dairy consumption, no significant differences in muscle strength were found between those who consumed less than one serving per day and those who consumed one or more servings per day (*p* = 0.83). Average muscle strength was slightly higher in participants who consumed two or more servings of legumes or eggs per week compared to those who consumed less than two servings (17.5 ± 7.9 kg versus 16.8 ± 11.8 kg, respectively), although this difference did not reach statistical significance (*p* = 0.81). Similarly, participants with daily consumption of meat, fish, or poultry showed a higher average muscle strength compared to those who did not consume these foods daily (17.4 ± 7.8 kg versus 16.8 ± 9.6 kg), but the difference was not significant (*p* = 0.49). Although mean muscle strength was higher in individuals who consumed two or more servings of fruits and vegetables per day compared to those with lower consumption (17.8 ± 8.7 kg versus 15.9 ± 8.0 kg), this difference was not statistically significant either (*p* = 0.24). Finally, no significant differences in muscle strength were observed according to fluid intake (*p* = 0.93). With respect to muscle strength, no significant differences were found according to sex in most food groups, except between the average of muscle strength and fruit and vegetable consumption in women (*p* = 0.03). In addition, a trend was observed between average muscle strength and consumption of meat, fish and poultry in women; however, this trend was not significant.

Table 4 presents the distribution of muscle strength (normal vs. low) according to food consumption frequency. No significant associations were found between muscle strength level and the consumption of dairy products (*p* = 1.0), legumes or eggs (*p* = 0.37), meat, fish, or poultry (*p* = 0.2), or fluids (*p* = 0.61). However, a significant association was observed between muscle strength and the consumption of fruits and vegetables, with the consumption of two or more servings daily being more frequent in participants with normal muscle strength compared to those with low muscle strength (*p* = 0.02).

Table 5 presents the differences in muscle strength according to the categories of the protein index. Participants with a good protein index showed an average muscle strength of 18.1 ± 7.7 kg, while those with regular and low protein indices recorded averages of 16.7 ± 7.7 kg and 17.4 ± 11.0 kg, respectively. No statistically significant differences in muscle strength were observed between the protein index levels (*p* = 0.71). When conducting analyses according to sex, no significant difference was found in muscle strength or protein index.

Table 6 presents the association between muscle strength and various nutritional and sociodemographic variables. Significant associations were observed for fruit and vegetable consumption (OR = 4.28; 95% CI: 1.59–11.45; *p* = 0.004) and age (≥75 years vs. <75 years) (OR = 3.08; 95% CI: 1.42–6.68; *p* = 0.004). Participants whose daily consumption of fruits and vegetables was less than two servings were 4.28 times more likely to have low muscle strength compared to those who consumed two or more servings per day. Older persons aged ≥75 years had a 3.08 times higher risk of having low muscle strength compared to those aged <75 years. However, no significant associations were identified between muscle strength and the consumption of dairy products (OR = 1.38; *p* = 0.78), legumes or eggs (OR = 0.46; *p* = 0.47), or meat, fish, or poultry (OR = 0.92; *p* = 0.94). Furthermore, the protein index did not show significant associations for any of its categories (*p* > 0.05). Fluid consumption showed no significant relationship with muscle strength either. Participants consuming 3 to 5 cups daily (OR = 0.69; *p* = 0.59) or more than 5 cups daily (OR = 0.94; *p* = 0.93) did not show a different likelihood of having low muscle strength compared to those consuming less than 3 cups daily. Finally, no significant associations were observed with nutritional status as assessed by the MNA (OR = 1.17; *p* = 0.71). No significant associations were identified between muscle strength and gender (OR = 1.34; *p* = 0.48).

## 4. Discussion

This study explored the relationship between frequency of food consumption, evaluated through the MNA, and probable sarcopenia, as measured through grip strength, in a group of older Chilean persons aged 65 and over. The findings revealed that muscle strength did not show significant associations with most of the food groups analyzed, except for the consumption of fruits and vegetables. Specifically, participants who consumed less than two servings of fruits and vegetables per day were more likely to have low muscle strength, suggesting a possible protective role of these foods in preserving muscle function.

These results are in line with previous studies that have highlighted the importance of a diet rich in fruits and vegetables, which is associated with a lower risk of low muscle strength and the prevention of sarcopenia and/or its other criteria (low muscle mass and/or physical performance). A prospective cohort study examined the relationship between food groups consumption and changes in sarcopenia parameters over a year in 165 outpatient individuals aged 65 and older. Results showed that higher fruit consumption was associated with a lower risk of prolonged time on the five-times sit-to-stand test (5CST), indicating better physical function or performance. Additionally, a higher consumption of green and yellow vegetables decreased the risk of reduced gait speed [19].

Additionally, a study that evaluated the relationship between adherence to Mediterranean diet and grip strength in 2963 older persons from the Longevity Check-up 7+ project in Italy reported that greater adherence to Mediterranean diet was associated with a lower risk of probable sarcopenia. This association remained significant even after adjusting for age and sex [20].

The analysis stratified by sex found that muscle strength in women showed a greater association with the frequency of consumption of certain food products as compared to men. In women, a higher daily intake of fruits and vegetables was related to improved muscle strength, while the consumption of meat, fish and poultry showed a possitive trend in the bivariate analyses, without reaching statistical significance. However, such trend was not observed after multivariate analysis, suggesting that other factors may mediate this relationship. These results underscore the importance of considering the sex variable when studying the relationship between diet and muscle strength, and support the need for future research to explore the mechanisms involved.

A diet high in antioxidants and bioactive compounds, based on the consumption of plant-based foods like the Mediterranean diet, may play a crucial role in reducing oxidative stress and inflammation—key factors in the loss of muscle mass and function associated with aging [21,22]. The Mediterranean diet enhances the bioavailability of nitric oxide by incorporating foods that provide both substrates for nitric oxide synthase (NOS), such as L-arginine and nitrates, as well as positive NOS modulators, including vitamin C, polyphenols, and omega-3 fatty acids [23].

Our results did not show significant associations between muscle strength and the consumption of dairy products, legumes, eggs, meat, fish, or poultry, or fluids. Additionally, when assessing protein index, no statistically significant differences in muscle strength were found between the different protein index categories. These findings contrast with previous studies suggesting a positive association between protein intake and muscle strength in older persons.

Choi (2023) found that low protein intake was associated with a higher likelihood of decreased grip strength in Korean men over 60 years old, especially when they did not engage in regular strength exercises [24]. Similarly, Kim et al. (2023) reported that older women who consumed more protein than the recommended daily intake were less likely to have reduced grip strength [25].

Additionally, significant associations have been observed between protein intake and sarcopenia and/or its other criteria in the population over 60 years old. For example, a meta-analysis by Santiago et al. (2021) compared caloric and nutrient intake in older persons with and without sarcopenia, finding that those without sarcopenia consumed more protein, carbohydrates, fiber, vitamin B12, and iron [10]. Similarly, Han et al. (2024) conducted a systematic review and meta-analysis in older Korean persons, observing that lower protein intake was significantly associated with a higher risk of sarcopenia and decreased muscle strength [26].

Furthermore, another systematic review and meta-analysis examined cross-sectional and longitudinal associations between protein intake and sarcopenia in older persons. The meta-analysis of four studies indicated that older persons with sarcopenia consumed significantly less protein than their peers without sarcopenia [9].

From the perspective of food groups, the study by Takagi (2024) evidenced that higher legume consumption reduced the risk of decline in skeletal muscle mass index [19].

However, Robinson et al. emphasize that higher-quality diets, characterized by the frequent consumption of fruits, vegetables, whole-grain food products, legumes and fish, and a lower consumption of ultra-processed food, are associated with better outcomes in muscle function and the prevention of sarcopenia, since these dietary patterns are linked to a better preservation of muscle mass and muscle function [27].

The discrepancy between these results and those of our study could be explained by various methodological reasons, such as the use of the MNA as a dietary data collection tool. The complete version of the MNA was employed to conduct the nutritional assessment. Although this instrument considers the frequency of consumption of some protein sources, fruits and vegetables, water, and fluids, it does not allow an accurate quantification of servings consumed nor an assessment of other key food groups, such as cereals, oils, fats, and lipid-rich foods. This limits the ability to quantify protein intake, identify essential and critical nutrients, and analyze overall diet quality associated with malnutrition in older persons. Furthermore, the MNA does not allow for a detailed evaluation of specific dietary habits. However, the MNA is a validated tool for assessing nutritional status in older persons. It is cost-effective, reliable, non-invasive, and easy to use [28,29].

Chilean dietary consumption patterns have been characterized by a high consumption of bread and processed food products, and are associated with a higher proportion of overweight and obesity, particularly among lower socio-economic groups [30,31]. In Chile, data from the National Food Consumption Survey (ENCA 2010) [30] revealed that older persons aged 65 and older reported a low compliance with the dietary recommendations included in the Dietary Guidelines for the Chilean population. In this age group, only 19.4% met the recommendation for dairy products consumption (three servings of dairy per day), 13.1% met the recommendation for fish (twice a week), 29% met the recommendation for legumes (twice a week), 51.4% met the recommendation for fruits and vegetables (five servings of fruits and vegetables per day), and barely 13.2% met the recommended water intake (six to eight cups of water per day) [30].

Furthermore, diet quality may be affected by economic factors. The same survey showed that people from low socio-economic groups reported a lower consumption of fruits, vegetables, fish, and dairy products, representing the group that has the highest percentage of non-compliance with the Dietary Guidelines for the Chilean population [30].

Therefore, the findings of this study should be interpreted with this nutritional and sociocultural context taken into account, since it may contribute to a greater vulnerability to sarcopenia in this population.

In the present study, grip strength measurement using a dynamometer was employed to assess muscular strength. According to the EWGSOP2, grip strength measurement is a key criterion for determining sarcopenia, as a low grip strength is a significant predictor of adverse outcomes such as prolonged hospitalizations, greater functional limitations, decreased quality of life, and increased mortality [8]. Grip strength measurement is a simple and cost-effective technique. To obtain accurate measurements, it is essential to use a calibrated portable dynamometer under controlled testing conditions and with appropriate reference data [8]. In this study, we used the Jamar dynamometer, which is widely validated and commonly used for grip strength measurement.

However, our study has some limitations. First, the cross-sectional design limits the ability to establish causal relationships between food consumption and muscular strength. Additionally, the sample was predominantly composed of women, which may affect the generalizability of the results to the male population. The sample was non-probabilistic and convenience-based, meaning that the results may not be extrapolated to the general population. Although both institutionalized and non-institutionalized older persons were included, more than 90% of the sample consisted of non-institutionalized individuals from a lower-middle socio-economic background, which could be representative of a large portion of the older population in Chile.

In this context, it is relevant to consider the role of nutritional support programs targeted at this population. In Chile, the Elderly Complementary Feeding Program (PACAM) is part of a set of preventive and recovery-oriented nutritional support activities that distribute micronutrient-fortified foods to older persons in primary healthcare facilities [32]. However, since this program does not have universal coverage, its reach may be limited. Furthermore, it has been shown that adherence to PACAM is low [33], which could affect its effectiveness in preventing malnutrition and sarcopenia. In 2022, the program and its products were reformulated to improve adherence and the profile of critical nutrients in the population [34]. Nevertheless, it is essential to evaluate strategies to improve the program’s coverage, accessibility, and acceptance, especially in low-income groups at greater risk of nutritional deterioration.

Finally, our findings highlight the importance of promoting an adequate intake of fruits and vegetables, along with strategies that optimize nutritional status to mitigate the risk of sarcopenia. In this context, longitudinal studies are needed to assess the long-term impact of dietary consumption and its relationship with the evolution of muscle strength in this population.

## 5. Conclusions

This study provides evidence on the relationship between food consumption and muscle strength in Chilean older persons. Although no significant associations were observed with most food groups, the insufficient intake of fruits and vegetables was associated with a higher likelihood of having low muscle strength, i.e., probable sarcopenia. These findings emphasize the importance of implementing nutritional strategies that promote a balanced diet to prevent muscle deterioration during aging, as well as strengthening public health policies aimed at encouraging higher fruit and vegetable intake in this population.

Future studies should address these relationships through longitudinal designs and more representative samples, also considering other determinants of muscle strength, such as physical activity and general health status. Furthermore, the role of nutrition professionals is key in designing and implementing personalized nutritional interventions aimed at optimizing the intake of essential nutrients and contributing to healthy and functional aging.

## Figures and Tables

**Table 1:** Sociodemographic characterization of the sample, according to sex.

Variable	Total Sample n = 155	Men n = 43 (27.7)	Women n = 112 (72.3)	*p*-Value
Age (years) ^‡^	77.1 ± 7.1	76.5 ± 7.1	77.4 ± 7.1	0.24 ^(1)^
<75 years	58 (37.4)	17 (39.5)	41 (36.6)	0.80 ^(2)^
≥75 years	97 (62.6)	26 (60.5)	71 (63.4)	
Weight (kg) ^‡^	68.8 ± 13.7	74.7 ± 13.6	66.6 ± 13.1	<0.01 ^(1)^
Height (m) ^‡^	1.53 ± 0.1	1.63 ± 0.1	1.49 ± 0.1	<0.01 ^(1)^
BMI (kg/m^2^) ^‡^	29.1 ± 5.2	27.9 ± 4.8	29.6 ± 5.3	0.08 ^(1)^
Nutritional status according to BMI ^†^				
Underweight	11 (7.1)	4 (9.3)	7 (6.3)	0.36 ^(2)^
Normal weight	62 (40)	21 (48.8)	41 (36.6)	
Overweight	45 (29)	7 (16.3)	30 (26.8)	
Obesity	37 (23.9)	11 (25.6)	34 (30.4)	
Nutritional Status according to MNA ^†^				
Normal	103 (66.5)	28 (65.1)	75 (67)	0.66 ^(2)^
Risk of malnutrition	44 (28.4)	12 (27.9)	33 (29.5)	
Malnutrition	8 (5.2)	3 (7)	4 (3.6)	
Arm circumference ^‡^	28.5 ± 3.9	28.2 ± 3.7	28.7 ± 3.9	0.25 ^(1)^
Calf circumference ^‡^	35.1 ± 3.4	35.2 ± 3.2	35.0 ± 3.5	0.68 ^(1)^
Muscle strength (kg) ^‡^	17.3 ± 8.6	25.4 ± 9.7	14.2 ± 5.6	<0.01 ^(1)^
Place of Residence ^†^				
Institutionalized	15 (9.7)	7 (16.3)	8 (7.1)	0.07 ^(2)^
Non-institutionalized	140 (90.3)	36 (83.7)	104 (92.9)	

BMI: Body Mass Index. Place of residence: Institutionalized = Residents of Long-Term Care Facilities for Older Persons; Non-institutionalized = Older Persons from Assisted Housing Facilities, Groups, Clubs, and Private Individuals. Values correspond to the following: ^†^ Number (%). ^‡^ Mean ± Standard Deviation. *p*-value corresponds to the following: ^(1)^ Student’s *t*-test; ^(2)^ Chi-square test.

**Table 2:** Characteristics of diet and frequency of food consumption as assessed using the MNA, according to sex.

Variable	Total Sample n = 155	Men n = 43 (27.7)	Women n = 112 (72.3)	*p*-Value
Appetite in the last three months ^†^ (n = 148)				
Has eaten much less	6 (4.1)	0 (0.0)	6 (5.7)	0.21 ^(2)^
Has eaten less	34 (22.9)	8 (18.6)	26 (24.8)	
Has eaten the same	108 (72.9)	35 (81.4)	73 (69.5)	
Feeding ability ^†^ (n = 147)				
Needs assistance	2 (1.4)	0 (0.0)	2 (1.9)	1.00 ^(2)^
Feeds independently, with difficulty	5 (3.4)	1 (2.3)	4 (3.8)	
Feeds independently, without difficulty	140 (95.2)	42 (97.7)	98 (94.2)	
Number of full meals per day ^†^ (n = 155)				
1 or 2 meals per day	5 (3.2)	0 (0.0)	5 (4.5)	0.32 ^(2)^
3 meals per day	150 (96.8)	43 (100)	107 (95.5)	
Dairy product consumption ^†^ (n = 155)				
<1 serving of dairy per day	62 (40)	18 (41.9)	44 (39.3)	0.77 ^(1)^
≥1 serving of dairy per day	93 (60)	25 (58.1)	68 (60.7)	
Legume or egg consumption ^†^ (n = 155)				
<2 servings of legumes or eggs per week	22 (14.2)	8 (18.6)	14 (12.5)	0.33 ^(1)^
≥2 servings of legumes or eggs per week	133 (85.8)	35 (81.4)	98 (87.5)	
Meat, fish, or poultry consumption ^†^ (n = 155)				
Does not consume meat, fish, or poultry daily	64 (41.3)	19 (44.2)	45 (40.2)	0.65 ^(1)^
Consumes meat, fish, or poultry daily	91 (58.7)	24 (55.8)	67 (59.8)	
Fruit and vegetable consumption ^†^ (n = 155)				
<2 servings of fruits or vegetables per day	36 (23.2)	12 (27.9)	24 (21.4)	0.39 ^(1)^
≥2 servings of fruits or vegetables per day	119 (76.8)	31 (72.1)	88 (78.6)	
Fluid intake ^†^ (n = 155)				
<3 cups of fluids per day	15 (9.7)	4 (9.3)	11 (9.8)	0.96 ^(2)^
3 to 5 cups of fluids per day	59 (38.1)	17 (39.5)	42 (37.5)	
>5 cups of fluids per day	81 (52.3)	22 (51.2)	59 (52.7)	

Values correspond to the following: ^†^ Number (%). *p*-value corresponds to the following: ^(1)^ Chi-square test; ^(2)^ Fisher’s exact test.

**Table 3:** Muscle strength and frequency of food consumption, according to sex.

Variable	Muscle Strength	*p*-Value	Men	*p*-Value	Women	*p*-Value
Dairy products consumption (n = 155)						
<1 serving of dairy per day	17.2 ± 8.3	0.83 ^(1)^	25.4 ± 7.8	0.99 ^(1)^	13.8 ± 5.8	0.48 ^(1)^
≥1 serving of dairy per day	17.5 ± 8.8		25.4 ± 11.1		14.5 ± 5.5	
Legume or egg consumption (n = 155)						
<2 servings of legumes or eggs per week	16.8 ± 11.8	0.81^(1)^	23.88 ± 6.17	0.77 ^(1)^	12.79 ± 3.53	0.15 ^(1)^
≥2 servings of legumes or eggs per week	17.5 ± 7.9		25.80 ± 7.29		14.45 ± 5.79	
Meat, fish, or poultry consumption (n = 155)						
Does not consume meat, fish, or poultry daily	16.8 ± 9.6	0.49 ^(1)^	25.58 ± 2.84	0.94 ^(1)^	13.07 ± 4.55	0.05 ^(1)^
Consumes meat, fish, or poultry daily	17.4 ± 7.8		25.33 ± 7.25		15.03 ± 6.07	
Fruit and vegetable consumption (n = 155)						
<2 servings of fruits or vegetables per day	15.9 ± 8.0	0.24 ^(1)^	23.67 ± 2.21	0.46 ^(1)^	12 ± 4.71	0.03 ^(1)^
≥2 servings of fruits or vegetables per day	17.8 ± 8.7		26.13 ± 1.87		14.85 ± 5.66	
Fluid intake (n = 155)						
<3 cups of fluids per day	17.1 ± 8.1	0.93 ^(2)^	26.0 ± 8.64	0.98 ^(2)^	13.91 ± 5.22	0.55 ^(2)^
3 to 5 cups of fluids per day	17.1 ± 7.9		25.65 ± 8.31		13.57 ± 4.53	
>5 cups of fluids per day	17.6 ± 9.1		25.18 ± 11.20		14.78 ± 6.30	

Values correspond to the following: Mean ± Standard Deviation. *p*-value corresponds to the following: ^(1)^ Student’s *t*-test; ^(2)^ ANOVA.

**Table 4:** Muscle strength level (normal vs. low) according to frequency of food consumption.

Variable	Muscle Strength		*p*-Value
	Normal n = 70	Low n = 85	
Dairy product consumption (n = 155)			
<1 serving of dairy per day	28 (40)	34 (40)	1.0
≥1 serving of dairy per day	42 (60)	51 (60)	
Legume or egg consumption (n = 155)			
<2 servings of legumes or eggs per week	8 (11.4)	14 (16.5)	0.37
≥2 servings of legumes or eggs per week	62 (88.6)	71 (84.5)	
Meat, fish, or poultry consumption (n = 155)			
Does not consume meat, fish, or poultry daily	25 (35.7)	39 (45.9)	0.2
Consumes meat, fish, or poultry daily	45 (64.3)	46 (54.1)	
Fruit and vegetable consumption (n = 155)			
<2 servings of fruits or vegetables per day	8 (11.4)	28 (32.9)	0.02
≥2 servings of fruits or vegetables per day	62 (88.6)	57 (67.1)	
Fluid intake (n = 155)			
<3 cups of fluids per day	5 (7.1)	10 (11.8)	0.61
3 to 5 cups of fluids per day	28 (40)	31 (36.5)	
>5 cups of fluids per day	37 (52.9)	44 (51.8)	

Values correspond to the following: Number (%). *p*-value corresponds to the following: Chi-square test.

**Table 5:** Relationship between muscle strength and protein intake index, according to sex.

Protein Index	Muscle Strength	*p*-Value	Men	*p*-Value	Women	*p*-Value
Good (n = 52)	18.1 ± 7.7	0.71	26.5 ± 6.7	0.83	15.5 ± 6.0	0.15
Regular (n = 66)	16.7 ± 7.7		24.4 ± 7.5		13.9 ± 5.6	
Low (n = 37)	17.4 ± 11.0		25.9 ± 14.3		12.8 ± 4.5	

Values correspond to the following: Mean ± Standard Deviation. *p* value corresponds to the following: ANOVA test.

**Table 6:** Association between low muscle strength and nutritional and sociodemographic variables based on logistic regression.

	Odds Ratio	95%CI	*p*-Value
Daily dairy intake (≥1 serving vs. <1 serving)	1.38	0.14–13.64	0.78
Weekly legumes or eggs intake (≥2 servings vs. <2 servings)	0.46	0.06–3.81	0.47
Daily meat, fish, or poultry intake (Yes vs. No)	0.92	0.09–8.75	0.94
Protein index (Ref: Low)			
Regular	0.59	0.04–8.55	0.69
Good	0.38	0.00–44.62	0.69
Daily fruit and vegetable intake (<2 servings vs. ≥2 servings)	4.28	1.59–11.45	0.004
Daily fluid intake (Ref: <3 cups)			
3 to 5 cups	0.69	0.17–2.70	0.59
>5 cups	0.94	0.25–3.55	0.93
Nutritional status according to MNA (Normal vs. Malnutrition)	1.17	0.52–2.65	0.71
Gender (Female vs. Male)	1.34	0.59–3.02	0.48
Age (≥75 years vs. <75 years)	3.08	1.42–6.68	0.004
Constant	3.25	0.31–34.21	0.33

Values correspond to odds ratios (OR) and 95% confidence intervals (95% CI) derived from logistic regression models, using low muscle strength (LMS) as the response variable. Predictor variables include nutritional and sociodemographic factors. The reference group for each variable is indicated in the table. The model has been adjusted for covariates such as gender, age (≥75 years vs. <75 years), and nutritional status according to the Mini Nutritional Assessment (MNA), daily fluid intake, and protein intake (dairy products, legumes/eggs, meat/fish/poultry). The constant represents the expected value of the outcome variable when all predictors are equal to zero.

## Data Availability

The data presented in this study are available upon request to the corresponding author. They are not publicly available as they contain personal health information.
